# Algorithm for Accurate CT Kidney Localization and Marking by Dual-polar Coordinates Multi-image Fusion

**DOI:** 10.2174/0115734056490490260901070801

**Published:** 2026-09-11

**Authors:** Yahui Peng, Chun Shi, Huiping Li, Xuquan Wen, Xianhua Dai

**Affiliations:** 1 School of Biomedical Engineering, Sun Yat-sen University, Shenzhen, 518107, P.R., China;; 2 School of Integrated Circuits and Information Engineering, Guangdong Polytechnic Normal University, Guangzhou, 510633, P.R., China;; 3 School of Electronics and Information Technology(School of Microelectronics), Sun Yat-sen University, Guangzhou, 510006, P.R., China;; 4 School of Cyber Science and Technology, Sun Yat-sen University, Shenzhen 518107, P.R., China

**Keywords:** Dual-polar coordinate system, Multi-image fusion, CT kidney localization, Kidney identification, Renal cysts, Spine localization

## Abstract

**Introduction:**

This work aims to address the challenges posed by the large number of kidney CT images, which reduce diagnostic efficiency. A multi-image fusion CT kidney localization and identification algorithm based on a dual-polar coordinate system is proposed.

**Methods:**

First, the spine position is determined from the kidney CT image, and a polar coordinate system is established accordingly. The kidney's polar coordinates are then derived based on the spine's polar coordinate system. This two-stage polar representation-where the spine is first localized using one polar coordinate system and the kidney is subsequently described relative to it-is termed “dual-polar coordinates.” In addition, an adaptive multi-image identification algorithm is proposed that leverages changes in renal polar radius across multiple images-before and after adaptation-to identify kidney images accurately. Finally, when data volume is limited, this algorithm can be extended to automatically annotate the kidney region, effectively reducing label noise and improving diagnostic efficiency.

**Results:**

Experimental results demonstrate the high accuracy of the proposed algorithm in spine and kidney localization, recognition, and annotation. It performs particularly well on kidney images with cysts, correctly identifying both normal and cystic renal tissues and providing complete kidney outlines.

**Discussion:**

By simplifying kidney localization *via* the dual-polar coordinate system and fusing information from multiple images, the algorithm effectively handles morphological variations caused by cysts. It reduces physicians’ workload and improves diagnostic robustness, offering a novel and efficient tool for medical imaging diagnosis.

**Conclusion:**

The proposed algorithm significantly enhances the accuracy and efficiency of kidney CT image analysis, especially for cyst-containing kidneys, and provides strong support for clinical diagnosis and treatment of renal diseases.

## 1 INTRODUCTION

In medical imaging diagnosis, Computed Tomography (CT), a non-invasive, high-resolution imaging technology, has been widely used to examine various organs of the human body. CT technology uses X-rays to tomographically scan the human body, receiving X-ray signals that penetrate the body through a detector and reconstructing them with a computer to obtain a clear tomographic image, with high resolution for both soft and hard tissues, and capable of accurately displaying the morphology and lesions of tissues such as muscles, bones, blood vessels, and so on. Especially in the diagnosis of diseases involving the skeletal, cardiovascular, and abdominal organs, CT offers unique advantages.

As an important organ in the human body, the kidneys' healthy state is crucial to maintaining the body's physiological balance. In the diagnosis of renal diseases, CT images provide rich anatomical and functional information. However, with the popularization of CT technology and improvements in imaging accuracy, patients often produce a large number of CT images during a single renal examination. In routine renal CT scanning, multiple protocols, such as plain and enhanced scanning, are used to acquire images with different contrast levels. Each scanning protocol may produce multiple images, and layer thickness settings and scanning range further affect the number of images. For example, in axial plane scanning, if the layer thickness is set to 3 mm and the scanning area covers the entire kidney, with a height difference between the right and left kidneys, the number of CT images containing the kidney may range from 40 to 50. These 40–50 images are multiple cross-sectional slices from the same person's kidney. However, the number of images per person is large; the total number of patients that a single hospital can collect is usually limited, which is far from sufficient for training deep learning models. Due to the diversity of kidney locations and morphology, physicians spend significant time browsing and analyzing these images during the diagnostic process, which not only increases their workload but may also reduce diagnostic efficiency.

Given the large number of CT images, quickly and accurately localizing kidney-only images has become an urgent problem. Traditional methods rely on doctors’ visual identification and empirical judgment, which is not only time-consuming and laborious, but also susceptible to subjective factors. To solve this problem, this work proposes a new algorithm for CT kidney localization identification based on multi-image fusion. The algorithm adopts the polar coordinate system, establishes the correlation relationship between the spine and the kidney, and uses consecutive multiple CT images to analyze and judge the shape change characteristics of the kidney, and automatically filters out the images containing the kidney. The algorithm provides the kidney locations and the corresponding CT picture file numbers in the stored file sequence. Shape change is chosen as the object of analysis because kidneys present unique shape features in CT pictures. By extracting these features and combining them with advanced image processing techniques, we achieve automatic identification and localization of kidney images. This algorithm not only provides a new and efficient method for medical imaging diagnosis but also shows important academic value and application prospects.

In summary, this work proposes a CT kidney accurate positioning and labeling algorithm based on dual-polar coordinate multi-image fusion to address the problem of a large number of kidney CT images and the low diagnostic efficiency of doctors, demonstrating the following innovations: First, the position information of the spine is described using polar coordinates. Then, the pole and polar radius of the kidney are determined along the spine's polar axis. The correlation between the abdominal spine and kidneys is deeply analyzed, and a dual-polar coordinate system is established to describe the coordinate relationship between the spine and kidneys. The innovative application of the adaptive multi-image discrimination algorithm to address the challenges posed by a large number of kidney images and changing morphology effectively improves diagnostic efficiency. It reduces misdiagnosis and omission, and at the same time, it realizes the automatic labeling of the kidney region, reduces manual intervention and data pollution, and provides powerful support for the accurate diagnosis and treatment of kidney diseases.

The contents of each section of this paper are briefly described as follows: Section 2 describes in detail the anatomical structures of the spine and kidneys and their CT cross-sectional morphological features, and analyzes the influence of renal cysts on the shape of the kidneys; Section 3 overviews the current status of research on kidney shape marking and localization techniques, and analyzes the strengths and weaknesses of the existing methods; Section 4 describes in detail the precise localization and marking algorithms for the CT kidney based on the dual-polar coordinates multi-map fusion specific implementation steps, including the spine and kidney localization algorithm; Section 5 verifies the accuracy of the algorithm in spine and kidney localization, identification and marking through experiments; Section 6 summarizes the main research content and results of this paper, and gives an outlook on the future research direction.

## 2 BACKGROUND

In medical imaging, renal shape marking and localization are of great significance, serving as the basis for diagnosing renal diseases and providing key information for surgical and radiation treatment planning. With the continuous advances in medical imaging and computer technologies, research on kidney shape marking and localization has achieved remarkable results. Among them, accurately labeling the kidney's horizontal cross-section is a key task in medical image analysis, especially crucial for diagnosing renal lesions (*e.g*., cysts, tumors). In the following, the renal shape recognition and segmentation annotation techniques, as well as localization techniques, will be introduced.

### 2.1 Kidney Shape Recognition and Segmentation Annotation Techniques

Imagingomics technology can extract large numbers of high-throughput imaging features from medical images, covering a wide range of aspects, including color, shape, and texture. By processing and analyzing these features, imagingomics can provide powerful guidance for clinical diagnosis and prognosis prediction. In renal disease research, imaging and histological methods have been widely used to extract and analyze renal shape features, opening a new approach to the diagnosis and treatment of renal diseases [1-3].

For the kidneys and surrounding organs in CT images, previous literature [4, 5] used the *a priori* knowledge of kidney location to select the kidney region of interest using the ribs and vertebrae as boundary markers, then removed the muscle and fat tissues with low gray value in the kidney region of interest using OTSU threshold segmentation, and finally segmented the kidney images of different types using different methods, which can realize semi-automatic kidney, liver and other abdominal organs synchronized annotation, improving annotation efficiency and accuracy. The improved scheme in previous studies [6-8] enhances segmentation robustness by filling voids *via* morphological closure operations, thereby reducing reliance on gray-threshold segmentation to extract the kidney contour. Still, it is susceptible to interference from low-density lesions such as cysts or lipomas.

As reported in a previous study [9], the thin walls and smooth edges of renal cysts make them easily distinguishable from normal renal tissues in cross-section. Still, multiple cysts may distort the overall kidney morphology, blurring the labeled boundaries. Studies [10, 11] significantly improved the labeling accuracy of cyst and tumor boundaries by fusing the anatomical clarity of CT with the soft-tissue contrast of MRI. Researchers [12] investigated the discriminative value of energy-spectrum CT plain single-energy images combined with histogram texture analysis for the differentiation of parapelvic cysts from hydronephrosis. Previous studies [13-15] have proposed a closed-operation algorithm with adaptive structural element sizes to dynamically adjust filter parameters for different lesions (*e.g*., cysts, calcifications), thereby avoiding over-segmentation of boundaries caused by traditional fixed parameters.

Kidney shape recognition and segmentation are the basis of kidney shape recognition, and the two functions are usually integrated. By processing and analyzing kidney CT images, the shape features of the kidney, such as long and short diameters, area, and volume, can be extracted, thereby enabling kidney shape recognition. Based on kidney shape recognition, the above algorithms have distinct advantages and disadvantages in terms of segmentation accuracy and efficiency. Still, all of them can achieve automatic kidney segmentation to a certain extent. With the development of deep learning, kidney segmentation algorithms have gradually become a research hotspot. These algorithms can automatically learn kidney shape and texture features through deep learning, thereby achieving more accurate segmentation.

A study [16] proposed constructing an atlas set of existing data, including images and segmentation results/labels, and then using an alignment algorithm to align the atlas images into the target image space to obtain a segmentation estimation of the target image. A 3D U-Net convolutional neural network model was further used for the segmentation and quantitative measurement of renal solid occupancy in abdominal enhanced CT images to achieve highly accurate renal shape labeling [17-19]. Earlier work [20-22] has further reviewed the application and research progress of Artificial Intelligence (AI) in Renal Cell Carcinoma (RCC) pathology image analysis. A wide range of AI applications in RCC pathology image analysis, including detection, grading, staging, typing, and tumor prognosis prediction, were explored, demonstrating that AI technology provides pathologists with a more accurate and efficient auxiliary diagnostic tool. Challenges in this field, such as the highly heterogeneous nature of RCC, limited data sizes, the generalization ability of models, and the interpretability of AI algorithms, are highlighted, and future development directions are proposed.

Shape recognition and segmentation technology can not only help doctors more intuitively understand kidney morphological changes, but also provide important references for diagnosing and treating kidney diseases. For example, in diagnosing renal tumors, comparing the tumor's shape with that of the normal kidney can help doctors localize and qualitatively diagnose the tumor.

When the amount of data is insufficient for AI training, establishing the positional formulae for the spine and kidneys using polar coordinates based on the abdominal positions of the spine and kidneys is a starting point. In this work, we primarily establish the positional formulae for the spine and kidneys in dual-polar coordinates based on the graphical correlation of abdominal internal organs.

### 2.2 Renal Localization Techniques

Kidney localization is the process of determining the exact location of the kidney in a CT image. At present, a variety of kidney localization algorithms have been proposed, including template-matching, feature-point, and machine-learning approaches. These algorithms can automatically localize kidneys to a certain extent. With advances in medical imaging technology and improvements in computational power, three-dimensional reconstruction technology has been increasingly applied to kidney localization. Through three-dimensional reconstruction, the spatial location and morphological changes of the kidneys can be visualized, providing doctors with more comprehensive diagnostic information.

It has been reported that the fat component of such tumors showed significant hypointensity on CT images, and additional marking of the tumor boundary was required to delineate and quantify its spatial relationship with the vertebral poles to assess the occupying effect [7, 8]. A three-dimensional coordinate system was established by extending the traditional two-dimensional polar coordinates into three dimensions along the longitudinal axis of the spine, thereby enabling joint analysis across multiple planes [23-26].

For CT image kidney recognition, there are many algorithms, demonstrating the rich results of the current field of medical image processing in kidney-related research. In terms of kidney shape recognition and segmentation annotation technology, it covers a variety of technical means from traditional methods to AI-based methods, including image histology methods, automatic segmentation based on prior knowledge, manual and semi-automatic annotation, optimization of segmentation by a variety of image processing techniques, as well as methods based on mapping and deep learning, which provide powerful technical support for the diagnosis and treatment of renal diseases. In terms of renal localization technology, multiple localization algorithms and the application of 3D reconstruction techniques are introduced further to enhance the accuracy and comprehensiveness of renal localization.

Although existing studies have made significant progress in kidney shape marking and localization, there remain challenges. For example, the large number of renal CT images with variable morphology may lead to errors in traditional localization methods when dealing with complex cases; existing algorithms may have difficulty accurately identifying and labeling cyst regions in renal images containing lesions, such as cysts.

To address these challenges, this paper proposes a new algorithm for accurate CT kidney localization and identification based on dual-polar coordinate multi-graph fusion, with significant innovations. Firstly, by introducing a polar coordinate system, a model of the spinal polar coordinate system was analyzed and established. Then, based on the positional relationship between the spine and the kidneys, a polar coordinate formula was established on the spinal polar axis to determine the position of the kidneys. The kidney positioning process was simplified using dual polar coordinates. Secondly, this study proposes an adaptive multi-image discrimination algorithm that uses the change in the renal polar diameter between the adaptive anterior and posterior images to accurately identify kidney images, effectively addressing the large number of kidney CT images and changing morphology. Finally, the algorithm can automatically label the kidney region, reducing the need for manual intervention and data contamination, and improving diagnostic efficiency. These innovative points not only offer new ideas and methods in medical imaging diagnosis but also provide strong support for the accurate diagnosis and treatment of kidney diseases.

## 3 RELATED WORK

Based on previous literature [27-29], this section presents an analysis of CT cross-sectional shape information of the spine and kidney to provide innovative ideas and rationale for the accurate determination and labeling of renal cysts.

### 3.1 Horizontal Cross-section Analysis of the Spine

#### 3.1.1 Overall Form and Function of the Spine

The spine, as the central axis of the human body, consists of multiple vertebrae connected to one another, and it is not only a key structure for supporting body weight and protecting the spinal cord and internal organs but also performs important functions such as transmitting pressure, cushioning vibration, and participating in body movement. When viewed from the back, the spine appears as a straight line. At the same time, the side shows a unique “S”-shaped curvature, including four physiological curvatures: cervical lordosis, thoracic kyphosis, lumbar lordosis, and sacral kyphosis, which help maintain the body's balance and stability.

Each vertebra consists of a vertebral body at the front and an arch at the back. The vertebral body is the main weight-bearing part of the vertebra, filled with cancellous bone and covered with dense bone. The arch consists of a bony plate with multiple projections (spinous processes, transverse processes, articular processes, *etc*.), which, together with the vertebral body, enclose the vertebral foramen. The maximum cross-sectional diameter of the vertebrae below the chest in adults, especially the lumbar vertebrae, varies with individual differences, age, gender, *etc*., and usually ranges from 20 to 30 millimeters. The lumbar vertebrae are particularly prominent in the spine, with their kidney-shaped cross-sections and stout physique, and the transverse diameter of the 3rd lumbar vertebra can reach approximately 46 mm in some individuals.

#### 3.1.2 Morphological Characterization of Horizontal Cross-Sections of the Lumbar Spine

The kidneys are located in the superficial fossa on both sides of the retroperitoneal spine and are symmetrically distributed between the left and right sides of the body. Specifically, the left kidney extends from the lower edge of the 11th thoracic vertebra to the intervertebral disc between the 2nd and 3rd lumbar vertebrae; the right kidney is located between the upper edge of the 12th thoracic vertebra and the upper edge of the 3rd lumbar vertebra. Given this anatomical relationship, the vertebral region containing the kidneys is primarily considered the lumbar spine.

The horizontal cross-section of the lumbar vertebrae is morphologically distinctive, with an overall appearance that usually approximates a circular or oval shape. In terms of specific dimensions, the lumbar vertebral body has a relatively large anterior-posterior height and a relatively small left-right width, resulting in a clear difference between the two. Moreover, this dimension ratio feature is not fixed across different segments of the lumbar vertebrae; rather, there is a pattern of change. For example, from lumbar spine 1 to lumbar spine 2, the cross-sectional shape of the vertebral body gradually tends to be close to a kidney shape; the cross-sectional shape of lumbar spine 3 or lumbar spine 4 may be closer to an ellipse; and the cross-sectional shape of lumbar spine 5 may be more special, and it may show an olive shape or other irregular shapes.

CT imaging is more focused on bony structures and provides a more accurate representation of the lumbar vertebrae's bone structure and density. The bone marrow cavity is located in the anterior-posterior center of the vertebral body, giving the lumbar spine a “hollow” circular or oval cross-section, with a core area surrounded by a bony annulus composed of the pedicle and the pedicle plate. These morphological features provide rich geometric parameters and a structural basis for the mathematical modeling of the lumbar vertebral cross-section, thereby enabling the construction of a more accurate and effective model.

### 3.2 Renal Morphology and Anatomical Morphology in Cross-Section at the CT Level

The kidney plays a vital role in maintaining the body's homeostasis and has a unique shape with distinctive features. The kidney, as a whole, is shaped like a fava bean and is reddish-brown. In terms of specific shape parameters, the kidney resembled a lentil, measuring 10-12 cm in length, 5-6 cm in width, 3-4 cm in thickness, and weighing 120-150 grams. The kidney's surface was smooth, with a slight bulge at the outer edge and a depression at the middle of the inner edge. The surface of the kidney is smooth, with a slightly elevated outer edge and a depression in the middle of the inner edge, which is the renal hilum, the passageway for the renal blood vessels, ureters, nerves, and lymphatics, and whose location and structure are critical to the realization of renal function. In the horizontal cross-section of a CT image, the kidney exhibits specific morphological features that are valuable for clinical diagnosis. Normally, the kidneys appear as round or oval soft-tissue densities with smooth, sharp edges on CT images, with occasional multiple cuts or localized convexity of the renal parenchyma.

In terms of kidney cross-sectional morphology, as it approaches the upper and lower poles, the kidney becomes more rounded. In contrast, at the level of the renal hilum, its inner side is concave to form the renal hilum. When the kidneys are in a pathological state, such as with renal cysts or tumors, they may appear irregular or exhibit localized bulging. Therefore, when analyzing CT images, doctors need to consider the overall morphology of the kidney, its internal structure, and dynamic changes after enhancement scanning to ensure accurate identification and diagnosis of the kidney, which also provides a key imaging basis for subsequent mathematical modeling based on the kidney's morphological features.

### 3.3 The Effect of Renal Cysts on the Appearance of the Kidneys

Renal cysts are common structural abnormalities of the kidneys, and their incidence increases with age. With advances in medical imaging, renal cysts have received increasing attention in diagnostic imaging. Renal cysts are mainly categorized into simple renal cysts and hereditary renal cysts based on etiology and clinical manifestations.

(1) Simple renal cysts is the most common type of renal cyst. In terms of appearance, it presents as a single or multiple cystic lesions. These cysts will make the kidneys appear localized and raised, changing the original smooth surface morphology of the kidneys. When the cysts increase in size or compress the surrounding tissues, they cannot only further change the shape of the kidney but also cause symptoms such as lower back and abdominal pain, an abdominal mass, and hematuria.

(2) Polycystic kidney disease is a common hereditary renal cystic disease. On appearance, multiple cysts of varying sizes are scattered bilaterally in the kidneys, increasing their overall size and making them irregularly shaped. Over time, the cysts enlarge progressively, and the normal structure and shape of the kidneys are more seriously damaged, resulting in impairment of kidney structure and function. Early patients may have no obvious symptoms, but as the disease progresses, the irregularity of the kidney's shape increases.

The Bosniak grading system is an important clinical tool in the imaging evaluation of renal cysts. It categorizes renal cystic masses into four grades based on CT or MRI to assess malignancy and guide clinical management. This system helps to differentiate between benign cysts and potentially malignant cystic masses, avoiding unnecessary surgical intervention.

Overall, renal cysts significantly alter kidney shape, ranging from localized bulging in simple renal cysts to an overall enlargement and irregular shape in polycystic kidneys. The shape of the kidney in cystic lesions changes with some regularity, but it is unpredictable due to the diversity of renal cysts and individual differences. Imaging histology studies have deepened the understanding of the pathophysiological mechanisms of renal cysts and provided scientific evidence and technical support for clinical identification, treatment strategy development, and prognosis assessment.

## 4 METHODS

### 4.1 Modeling and Localization Algorithms for Spine CT Image Analysis

#### 4.1.1 Spine CT Images With a Polar Coordinate System Parameter System

In the relevant CT images of the spine, the spine appears extremely complex and is not simply elliptical. Firstly, the key coordinate points of the spine are analyzed from a single CT image to obtain the spine's key coordinate points and calculate the coordinate information of the spine poles; then, the existence of the spine poles is determined by combining the judgment across consecutive images. A localized CT spine cross-section is given in Fig. (**1**).

As shown in Fig. (**1**), the spine morphology is affected by many factors, including the oval cross-section of the lumbar vertebrae, the “hollow” character of the bone marrow cavity, and the pattern of morphological change across different segments. To accurately locate and analyze vertebrae, we introduce the polar coordinate system (*ρ,θ*) to construct an analytical model for vertebral images. The polar coordinate system can more flexibly describe the position and morphology of the spine in the image, providing a powerful mathematical tool for the design of subsequent algorithms.

To accurately characterize the position and morphology of the spine in the CT image, four key coordinates (*P_U_*, *P_D_*, *P_L_*, *P_R_*) are defined, which are the uppermost coordinate *P_U_* (*ρ_U_*,(*x_U_*_,_ *y_U_*), *θ_U_*), the lowermost coordinate *P_D_* (*ρ_D_*,(*x_D_*_,_ *y_U_*), *θ_D_*), the leftmost coordinate *P_L_* (*ρ_L_*,(*x_L_*_,_ *y_L_*), *θ_L_*), and the rightmost coordinate *P_R_* (*ρ_R_*,(*x_R_*_,_ *y_R_*), *θ_R_*). These coordinates will help us to initially determine the approximate extent of the spine in the image and provide the basis for subsequent pole calculations.

#### 4.1.2 Determination of Key Coordinates

(1) The uppermost coordinate *P_U_* was determined: in the horizontal-plane CT image, the middle and lower 1/3 of the region were analyzed, with the vertical line at the center of the image. Taking *θ* = 90° (vertical direction), the white boundary of a high-density signal band (CT value ≥ 800 HU) of 5 or 7 consecutive pixels was selected (with the inner border preferred).

(2) Allow a left-right offset of the boundary of ≤ 4 cm, while the trend of this white boundary direction is toward the bottom. Find the location where *ρ* takes the maximum value *ρ_U_,* which is the uppermost coordinate of the spine in the vertical direction.

(3) Determination of the *P_D_* of the lowermost coordinate: Taking *θ* = 90°, in the same analyzed area, referring to the way of finding the uppermost coordinate, *ρ* takes the position of the smallest value, | *ρ^Y^_U_ - ρ^Y^_D_* | ≤ 6*cm*, and this position is the lowermost coordinate. Through the uppermost and lowermost coordinates, the range of the spine in the vertical direction can be preliminarily determined, which provides an important basis for the subsequent determination of the pole.

(4) The leftmost coordinate *P_L_* was determined by first determining *ρ^Y^_L_* = (*ρ^Y^_U_* + *ρ^Y^_D_*)/2. From the position of *ρ^Y^_L_*, it was extended to the left (*θ* = 180°) until it touched the brightest boundary (usually the portion of the vertebral arch or bone cortex with a CT value of ≥ 800 HU), and the value at this point was recorded; this position was the leftmost coordinate. The leftmost coordinate helps determine the left boundary of the spine in the horizontal direction, and given the relatively small width of the lumbar vertebral body in the left-right direction, determining the leftmost point is important for accurately describing spinal morphology.

(5) Rightmost Coordinate *P_R_* Determination: similarly, first determine *ρ^Y^_L_* = (*ρ^Y^_U_* + *ρ^Y^_D_*)/2. Extend the vertebra from the position of *ρ^Y^_L_* to the right (*θ* = 0°) until it touches the brightest boundary, record the value at this point, and that position will be the rightmost coordinate. The rightmost coordinate determines the right boundary of the spine in the horizontal direction.

In spine CT image analysis, the four key coordinates, the uppermost, lowermost, leftmost, and rightmost, define the approximate range of the spine in the image. The uppermost and lowermost coordinates determine the vertical distribution, while the leftmost and rightmost coordinates define the horizontal distribution. Together, these coordinates provide an important basis for the accurate determination of the subsequent poles. As the center of the spine, the poles are crucial for analyzing spinal morphology, structure, and spatial relationships with surrounding tissues, thereby providing key information for diagnosing spinal diseases, planning treatment, and navigating surgery. At the center of the spine, accurate pole calculation is crucial for analyzing its morphology, structure, and spatial relationships with surrounding tissues, providing key information for diagnosing spine diseases, formulating treatment plans, and guiding surgical navigation.

#### 4.1.3 **Algorithm for Determining the Vertebral Pole (**P_S_**)**

The determination of the vertebral pole provides a benchmark for subsequent, more precise analysis, representing the center of the vertebra in the CT image and helping establish the spatial relationship between the vertebra and surrounding structures (*e.g*., kidneys). The initial spine pole determination algorithm, based on the uppermost *P_U_* and lowermost *P_D_* coordinates, can determine the approximate location of the center of the corresponding oval of the spine, and the center of this circle is used as the new pole of the algorithm. Let *P_S_* (*ρ_S_*,(*x_S_*_,_ *y_S_*), *θ_S_*), describe the polar coordinate information of the vertebral pole, and let *θs* = 0, rotated in the counterclockwise direction to a positive value in the range of [0,2*π*), and *ρ_S_*,(*x_S_*_,_ *y_S_*) be the vertebral pole of this CT map.


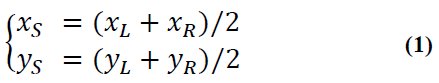


Equation (**1**) gives the initial coordinate information of the vertebral pole *ρ^I^_S_*(*x^I^_S_,* y*^I^_S_*). Due to inaccuracies in key coordinate data, the multi-plot calculation may cause the spine pole offset value to differ across plots, affecting the accuracy of the judgment. It is also necessary to correct the accurate information of the vertebral pole with *ρ^I^_S_* as the initial vertebral pole, *r^I^_S_* as the initial radius, and ∆ = 0.1cm as the error value.


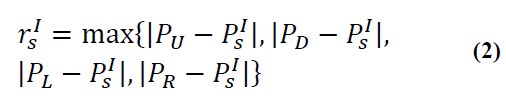


Equation (**2**) defines the initial transition radius *r^I^_S_* based on the maximum distance from the preliminary vertebral pole to the four boundary points. With *ρ^I^_S_* as the initial center and *r^I^_S_* as the initial radius, a circle was made around the inner boundary of the spine in the CT image (let *P^I^*(*ρ^I^*(*x^I^, y^I^*), *θ^I^*) be the corresponding point) (CT value ≥ 800 HU), and the distance between the pole and the inner boundary of the spine was *L^I^*:


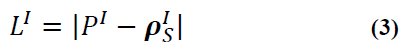


Equation (**3**) is then used to calculate the distance LI between the vertebral pole and the inner boundary point.

Let the error between *r^I^_S_* and *L^I^* be *δ^I^_S_* = *L^I^*- *r^I^_S_*. According to the magnitude of the error value *δ^I^_S_*, the pole position needs to be adjusted as follows:


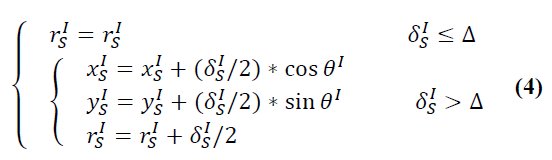


In Equation (**4**), when the distance between the temporary vertebral pole *ρ^I^_S_* and the vertebral inner boundary is less than the transition radius *r^I^_S_*, the pole position is not adjusted. When the distance between the vertebral pole *ρ^I^_S_* and the inner boundary of the spine is greater than the transition radius *r^I^_S_*, half of the error *δ^I^_S_* is used as the hypotenuse, and the pole position is adjusted according to the triangular relationship. The length of the transition radius is also adjusted.

An iterative algorithm is used to form new transition poles and transition radii by continuously adjusting the positions of the transition poles so that all vertebrae can be contained within a circle defined by the transition poles and radii, thereby determining the vertebral poles. After the iterative correction, the final vertebral pole is obtained using Equation (**5**).


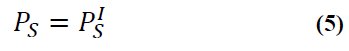


The above gives the procedure for determining the vertebral poles in a single CT image. Since there are multiple possible similar structures in the CT image, it is also necessary to determine the validity of the vertebrae and poles.

#### 4.1.4 Spine Image Existence Determination and Pole Passing

(1) Judgment of the existence of spine images: in the process of determining the vertebral poles, it is proposed to use a similar information fusion of consecutive multiple images to make the judgment. Given the continuity of vertebral appearances, the consistency of the same location across multiple images can support logical judgment in determining the presence of vertebrae in CT images. Specifically, it is no longer limited to calculating the pixel density, gray gradient, and other features in the region of the spine within a single image and comparing them with a preset threshold, but rather comprehensively analyzing the changes in the values of the poles and radii of the corresponding locations in consecutive multi-images, as well as combining the morphological features of the spine, to make a comprehensive judgment. If the poles and radii exist at similar positions from multiple images, the spine is judged to exist in the segment of consecutive images; otherwise, it is judged not to exist.

(2) Determination of poles in subsequent images: Based on the above logic of judging the existence of vertebrae by fusion of consecutive images, the pole position of the previous image is no longer used as a direct reference for the determination of poles in subsequent images. Since the position and morphology of the spine may change across pictures, each picture still needs to be analyzed independently for preliminary analysis. Still, it can rely on the fusion information from multiple images that have already been used to judge the spine's existence in the previous one. When dealing with continuous image sequences, the tracking algorithm is used to combine the approximate position of the spine obtained from the fusion of the previous multiple images, the movement trend, and the current frame of the image’s own characteristics, to predict and correct the poles of the subsequent frames, to improve the efficiency and accuracy of the determination of the poles, and to ensure the robustness of the algorithm in dealing with the continuous image sequences.

For clarity, the complete procedure of the spine pole localization and existence determination is presented in Fig. (**2**).

The spine CT image analysis, modeling, and localization algorithm initially determines the spine range by introducing a polar coordinate system to define four key coordinates. The pole position is then iteratively calculated by fusing the data. The spine's existence is determined using consecutive multi-image similarity and morphological features, and the subsequent tracking algorithm predicts the corrected pole by combining multi-image information, thereby improving the efficiency and accuracy of consecutive image sequence processing and providing a solid foundation for subsequent medical analysis and diagnosis.

### 4.2 Renal CT Image Localization Algorithm and Annotation Algorithm for the Identification of Cyst-Containing Kidneys

#### 4.2.1 Kidney Horizontal Cross-Sectional Shape Analysis

In a horizontal cross-section, the middle portion of a normal kidney is approximately circular or oval, centered on the renal hilum. In a horizontal cross-section from top to bottom, the kidney gradually increases in size until reaching a maximum and then decreases.

#### 4.2.2 Horizontal Cross-Sectional Shape Analysis of Cyst-Containing Kidneys

Renal cysts significantly alter the shape of the kidney's horizontal cross-section. Depending on the size, number, and location of the cysts, the renal cross-section may take on a different shape:

(1) Renal cysts are located in the upper part of the kidney; small cysts may have no obvious effect. In contrast, large cysts cause the upper portion of the kidney to bulge and become irregularly shaped, with the edges of the bulging portion smooth and well demarcated from the surrounding renal tissue.

(2) Renal cysts located in the middle of the kidney: large cysts bulge the middle cross-section of the kidney; the upper and lower parts of the kidney may be deformed due to the extrusion of the cysts, and the overall shape is irregular.

(3) Renal cysts are located in the lower part of the kidney. Large cysts bulge the lower pole in cross-section, and the upper pole may be displaced upward due to the extrusion of the cyst, resulting in an overall irregular shape.

(4) Multiple renal cysts: cysts compress one another, resulting in severe distortion of the kidney's cross-sectional shape and unevenness.

(5) The effect of renal cysts on kidney position: sufficiently large renal cysts may cause the kidneys to deviate from their original positions, disrupting renal image symmetry.

Renal cystic lesions often do not exhibit a highly defined or regular pattern of outer borders of the cystic portion of the cyst on CT images. Specifically, there may be localized blurring and discontinuity in the borders of the cyst. This may be related to several factors, including the cyst's growth characteristics, infiltration of the surrounding tissue, and CT imaging resolution and scanning parameters. When interpreting CT images, it is necessary to combine the patient’s clinical manifestations and other imaging features to perform a comprehensive analysis and accurately determine the nature of the cyst.

#### 4.2.3 Renal Pole Confirmation Algorithm

Usually, the kidneys are located on the left and right sides of the spine; here, the left side is used as an example for the detailed description of the algorithm. For cyst-containing kidney images, confirmation of the kidney pole *P_T_* serves as the basis for subsequent analysis and modeling.

Figure **3** shows a schematic diagram of the correlation between spine poles and kidney poles. Combined with the above schematic diagram, the detailed steps of the renal pole *P_T_* (*ρ_T_*,(*x_T_*_,_ *y_T_*), *θ_T_*) confirmation algorithm are as follows:

(1) Selection of renal and vertebral pole tangents

The solution is obtained by knowing the vertebral pole *P_s_* (*ρ_S_*,(*x_S_*_,_ *y_s_*), *θ_s_*), choosing the suspected kidney boundary point *Z* (*x_Z_*_,_ *y_Z_*), and connecting *P_s_* and *Z* to form the straight line *L_SZ_: Ax + By + C =* . The coefficients of the line *L_SZ_* are calculated using Equation (**6**).


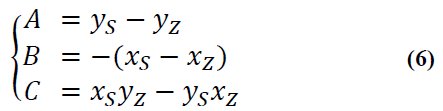


The linear *L_SZ_* angle (0° on the X-axis, positive counterclockwise) is in Equation (**7**):


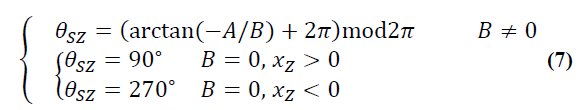


Moving the point Z yields the maximum and minimum angles (*θ_SZ_^MIN^*, *θ_SZ_^MAX^*) of *θ_SZ_*, which in turn determines the upper tangent *L*_2_ (feature point *P*^2^ (*ρ*_2_,(*x*_2,_ *y*_2_), *θ*_2_)) corresponding to the minimum angle *θ_SZ_^MIN^*, and the lower tangent *L*_1_ (feature point *P*^1^ (*ρ*_1_,(*x*_1,_ *y*_1_), *θ*_1_)) corresponding to the maximum angle *θ_SZ_^MAX^* of feature point *P*^2^.

During the movement of point Z, the point furthest from the vertebral pole and the nearest point *Z_N_* (*ρ_N_*,(*x_N_*_,_ *y_N_*), *θ_N_*) are kept.

It is proposed to assist in accurate kidney contour recognition by quantifying the angular change of the line connecting the kidney's outer boundary and the vertebral pole. The method leverages the anatomical relationship between the kidney and the spine, incorporates spatial constraint information, and effectively mitigates interference from blurred boundaries. By quantifying the angular changes, the objectivity and repeatability of contour recognition are significantly improved.

(2) Vertical Intersection Calculation

Based on the two key feature points *P*^1^ and *P*^2^ determined in the previous step, the operation of establishing normals was carried out: at feature point *P*^1^, the normal corresponding to tangent line *L*_1_ was constructed based on the geometric property of the tangent line; similarly, the normal corresponding to tangent line *L*_2_ was constructed at feature point *P*^2^. These two normals intersect at a point named *P_T_^I^*, and the vertebral pole *P_S_* and the point *P_T_^I^* are connected to form a straight line *L*. Assuming the renal pole lies on the straight line *L,* the point *P_T_^I^*can be regarded as the initial localization of the renal pole.

This construction method cleverly leverages the tangential nature of the feature points and combines the positional information of the vertebral poles to achieve localization of the kidney poles. The approach successfully establishes a mapping relationship between local features (spinal and renal) and a global reference (the correlation between spinal and renal poles), providing an important geometric reference for subsequent related studies.

(3) Radius Determination and Pole Optimization

Let the point *P_T_^I^* be the initial center of the kidney image and *r_T_^I^* be the initial radius. The radius value is determined as follows:


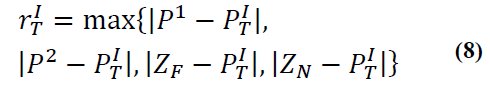


The initial renal radius *r_T_^I^* is determined using Equation (**8**). Given the irregularity of kidney shape, especially the cystic lesion kidney structure, based on the initial kidney pole *P_T_^I^* and the initial radius *r_T_^I^*, this paper restricts the kidney pole to move along the straight line *L* in Fig. (**2**), which in turn determines the appropriate radius. The adjustment strategy is as follows:

(3.1) If the radius, *r_T_^I^* = max{| *P*^1^- *P_T_^I^* |,| *P*^2^- *P_T_^I^*|}, indicates that the shape of the kidney presents a flattened oval in the direction of *P*^1^ and *P*^2^. Determine the kidney poles and radii as in Equation (**9**):


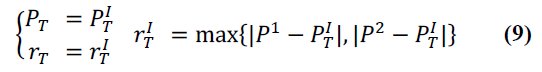


(3.2) If the radius, *r_T_^I^* = max{| *Z_F_*- *P_T_^I^* |,| *Z_N_*- *P_T_^I^*|}, indicates that the kidney shape is more distinctly distributed farther from the vertebral pole. Order *δ_T_^I^* = *r_T_^I^* - max{| *P*^1^- *P_T_^I^* |,| *P*^2^- *P_T_^I^*|}, Determine the kidney poles and radii as in Equation (**10**):


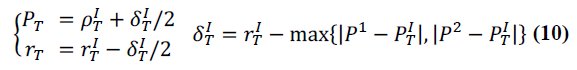


#### 4.2.4 Algorithm for Determining CT Kidney Images by Multi-image Fusion

After suspected kidney detection in a single CT image, the kidney must be accurately delineated using multi-map fusion. The following is a detailed algorithm for determining the CT kidney image by multi-map fusion with special emphasis on the role of polar coordinates in it:

(1) Confirmation of Single-Image Suspicions

In a single CT image, a preliminary circle is generated using the renal pole confirmation algorithm (as described in 4.2.3) if the kidney's starting position has not been clearly determined. If the majority of the renal region in the image (including possible cystic regions) is contained by the circle, the region is initially suspected to be a kidney.

(2) Judgment of the Correlation of Multiple Plots in Polar Coordinates

The kidney poles of each suspected kidney image were converted to representations under the polar coordinate system, and their radial distances ρ and angles θ were recorded. In the subsequent images, the kidneys were confirmed in the same way as in the single-image judgment, and their poles were converted to polar-coordinate representations. The polar coordinates of the suspected kidney poles in the two/multiple images before and after were compared, and the radial and angular variations ρ and ∆θ were calculated to determine whether the pole locations were correlated. If |∆ρ| *<* δ and |∆θ| *<* δ(δ is a preset threshold) are satisfied, the kidney poles in the two maps are considered to be correlated.

(3) Multi-Map Confirmation of Similar Regional Scope

Based on the polar coordinate correlation judgment, other images are further searched within the range of similar regions to identify regions with similar polar coordinate features and kidney morphological structures. If regions with similar polar-coordinate features and kidney morphology and structure are found across multiple images, they can be identified as kidneys.

(4) Determination of the Extent of Cystic Lesions

By comparing changes in kidney radius across images, the degree of cystic lesions can be preliminarily determined. By combining the changes in kidney pole position, radius size, and kidney morphology in multiple images, and according to “4.2.2 Analysis of horizontal cross-section shape of kidneys containing cysts”, we can judge the position and changes of cysts in kidneys, and comprehensively determine the degree of cystic lesions in kidneys, to provide an important basis for clinical diagnosis and treatment.

Using the above method, we can effectively identify kidney structures in images and distinguish normal kidneys from cyst-containing ones. This method combines single-image judgment and multi-image correlation judgment, thereby improving the accuracy and robustness of kidney recognition annotations.

For clarity, the complete procedure of the kidney pole confirmation and multi-image fusion identification is summarized in Fig. (**4**).

In this section, we describe in detail the methods for locating and marking the spine and kidney in the CT kidney, as well as an accurate localization and marking algorithm based on dual-polar coordinate multi-image fusion. By introducing the polar coordinate system, we can flexibly describe the position and morphology of the spine in CT images and precisely locate it through key coordinate determination and pole iteration. For the kidneys, we analyzed their morphological features in normal and cyst-containing states. We proposed a kidney-pole confirmation algorithm, which, when combined with a multi-image fusion strategy, improved the accuracy and robustness of kidney identification. The algorithm simplifies kidney localization, improves diagnostic efficiency, and offers a new approach to medical imaging diagnosis.

In summary, the algorithm in this section innovatively uses the polar coordinate system to correlate the spine with the kidneys, thereby achieving accurate localization and identification of the kidneys.

## 5 RESULTS

To comprehensively evaluate the effectiveness of the CT kidney accurate localization and labeling algorithm based on dual-polar coordinate multi-graph fusion proposed in this paper, we selected 38 cases from 129 medical records at the Affiliated Hospital of Sun Yat-sen University for validation. The validation has two main parts: in the first part, we validate the accuracy of the dual-polar coordinate multi-image fusion algorithm in identifying and labeling the kidneys; in the second part, we manually label the location of the renal cyst lesions of the 38 cases as the gold standard for evaluation, specifically including the start and end numbers of the corresponding image files of the renal cyst lesions and the file number of the largest area of the renal cysts. Then, the algorithm proposed in this paper was used to automatically identify the start and termination numbers of the kidney (including the cystic lesion), as well as the file number corresponding to the kidney's maximum area, to compare and validate against the gold standard.

### 5.1 Dual-polar Coordinates To Calibrate the Shape of the Spine and Kidneys

Validate the effectiveness of polar coordinates in spine localization and kidney localization and identification algorithms, and assess whether they improve the accuracy and efficiency of kidney identification. Implement a polar coordinate system for spine localization, kidney localization, and identification algorithms. A CT image dataset containing spine and kidney structures is selected. The spine localization, identification, and labeling algorithms were implemented as described in the document. The success rate and localization error of the algorithm for spine localization were calculated using the manually labeled spine position as the gold standard. The results are shown in Fig. (**5**).

Figure **5** presents a schematic diagram of the spine-kidney correlation and kidney shape calibration. The polar coordinate algorithm shows an advantage in processing speed, especially when handling a large number of images, and can more accurately identify cyst-containing kidneys despite changes in kidney shape.

The graph clearly shows the relative position of the spine pole and the kidney pole. This relationship aligns with the theory described in the algorithm for indirectly determining kidney location *via* spine localization. Specifically, the position and morphology of the kidneys in the graph are highly consistent with the results calculated by the algorithm, demonstrating the algorithm's accuracy in kidney localization and identification.

Furthermore, the graphics demonstrate the algorithm's effectiveness in processing images of cyst-containing kidneys. Due to the presence of cysts, the kidney's morphology may change. However, with the introduction of the polar coordinate system and the application of the multi-image fusion strategy, the algorithm can accurately identify the cyst region and distinguish it from normal kidney tissue. This capability is particularly important in practical clinical applications, as it helps improve the diagnostic accuracy and efficiency in kidney disease.

Polar coordinates play an important role in kidney localization and identification algorithms, improving their accuracy and efficiency and enhancing robustness to changes in kidney morphology.

The algorithm has a high success rate in kidney localization, especially when dealing with cyst-containing kidneys, showing significant advantages in accurately identifying the start and end positions of kidneys, as well as the file number of the kidney with the largest area, which provides key information for clinical diagnosis (Fig. **6**).

### 5.2 Verification of Renal Localization, Identification, and Marking Accuracy

To verify the accuracy of the algorithm in kidney localization, recognition, and identification, especially for cyst-containing kidneys, we selected a CT image dataset covering both normal and cyst-containing kidneys and strictly followed the algorithmic process described in the document for implementation. The success rate and localization error of the algorithm for kidney localization are calculated using the manually labeled renal cyst lesion as the gold standard, as shown in Fig. (**6**).

Further analysis reveals that the algorithm not only accurately distinguishes normal kidneys from cyst-containing kidneys but also completely outlines the kidneys and accurately identifies the cyst regions. Importantly, the range of file numbers calibrated by the algorithm overlaps with the manually calibrated numbers for renal cysts and includes the kidney without cystic lesions, reflecting the algorithm's comprehensiveness and superiority in identifying cyst-containing kidneys. In summary, the algorithm performs well in kidney localization, identification, and labeling, and injects new vitality into the field of medical imaging diagnosis.

The algorithm achieves high accuracy in kidney localization, identification, and labeling, especially for cyst-containing kidneys, with significant improvements, which contribute to the development of the medical imaging diagnosis field.

## 6 DISCUSSION

This work addresses a practical clinical challenge: a single patient typically produces dozens of kidney CT slices (*e.g*., 40–50), yet the total number of patients available at most hospitals is too small to train deep learning models from scratch. In this scenario, pure data-driven AI methods are prone to overfitting, while manual reading is time-consuming and susceptible to fatigue-induced errors. The proposed dual-polar coordinate multi-image fusion algorithm fills this gap by offering a model-driven, interpretable solution that leverages the stable anatomical relationship between the spine and kidneys. It automatically filters non-kidney slices, marks the kidney region, and provides clinical key information such as the start/end slice numbers and the slice with the largest kidney area. Experimental results confirm high accuracy even for cyst-containing kidneys, effectively reducing physicians’ workload and improving diagnostic consistency.

By simplifying kidney localization *via* the dual-polar coordinate system and fusing information from multiple images, the algorithm robustly handles morphological variations caused by renal cysts, including local bulging, shape irregularity, and even kidney displacement. Unlike conventional threshold-based or template-matching methods, the dual-polar representation and adaptive polar-radius change analysis do not require explicit cyst boundary segmentation. Yet, they successfully distinguish normal renal tissue from cystic lesions and provide complete kidney outlines. This robustness is particularly valuable in real-world clinical settings where cyst size, number, and location vary unpredictably.

Nevertheless, from the perspective of algorithmic precision and program implementation, further optimization is needed. The iterative pole determination and tangent-based kidney center estimation currently involve approximations (*e.g*., an error threshold Δ = 0.1 cm). In cases with extremely irregular cysts or severe kidney displacement, the polar radius adjustment may introduce minor localization errors. Future work will refine the iterative strategy-for instance, by incorporating adaptive convergence criteria or edge-preserving filtering-and improve computational efficiency through parallel processing or pre-filtering of irrelevant slices, aiming toward near-real-time clinical assistance.

We also recognize that artificial intelligence represents the future trend in medical image analysis. Although deep learning is not directly applicable due to limited patient data, the proposed algorithm can serve as a valuable prior or verification module for AI training. For example, its automatically labeled kidney regions can generate coarse annotations to reduce manual labeling effort, or the dual-polar coordinate relationship can be embedded into neural networks as an anatomical constraint to improve model interpretability and generalization. Moreover, the deterministic rules of this algorithm can act as a plausibility check for AI outputs, identifying unrealistic kidney locations and thereby enhancing clinical safety. In this sense, the algorithm not only stands as an efficient standalone tool but also paves the way for future hybrid systems that combine geometric reasoning with deep learning.

## CONCLUSION

In this study, we propose an accurate localization and identification algorithm for CT kidneys based on dual-polar coordinate multi-image fusion, aiming to address the challenges posed by the large number of kidney CT images and the low diagnostic efficiency of doctors. The innovations of the algorithm are mainly reflected in the following aspects:

(1) Expression method for polar-coordinate identification of kidney images: In this paper, we propose a method for identifying kidney images using a polar coordinate system and aim to standardize it. This method not only simplifies the description of kidney images but also facilitates subsequent processing and analysis. At the same time, by considering temporal information and image data from different stages of a single individual, the algorithm can better understand and analyze kidney morphological changes.

(2) Analysis of the spine-kidney correlation: The algorithm examines the correlation between the spine and the kidneys, indirectly determining the location of the kidney's cross-section by localizing the spine. This method uses the spine's anatomical characteristics as the central axis of the human body. It provides a new way of thinking about kidney localization.

(3) Adaptive multi-graph discrimination algorithm: In this paper, an adaptive multi-map discrimination algorithm is proposed that utilizes the magnitude of change in renal polar diameter in the adaptive before-and-after maps to accurately segment kidney images. This method can effectively handle kidney images with complex morphology, such as cysts, and improve the accuracy and efficiency of diagnosis.

(4) Automatic renal region annotation: the algorithm automatically annotates renal regions, reducing data contamination and the need for manual intervention. This not only improves diagnostic efficiency but also provides strong support for the accurate diagnosis and treatment of kidney diseases.

In the experimental validation section, this paper comprehensively evaluates the algorithm's effectiveness across three sets of experiments. The experimental results show that the algorithm achieves high accuracy in localization, recognition, and identification of both vertebrae and kidneys, and especially excels at processing cyst-containing kidney images. In addition, the algorithm shows significant advantages in processing speed and robustness.

In summary, the CT kidney accurate localization and marking algorithm based on dual-polar coordinate multi-image fusion proposed in this paper provides a new and efficient method for medical imaging diagnosis. The algorithm not only improves doctors' diagnostic efficiency but also provides strong support for accurate diagnosis and treatment of kidney diseases. In the future, we will continue to optimize the algorithm and explore its potential application in more medical imaging diagnosis scenarios.

## Figures and Tables

**Fig. (1):**
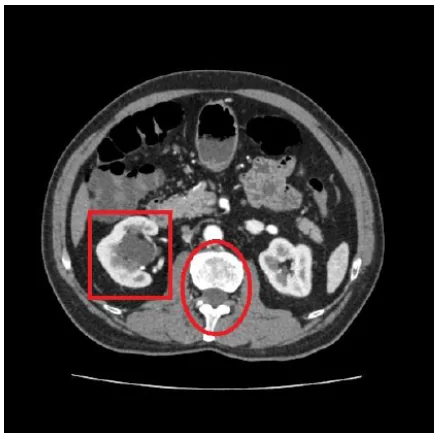
CT horizontal cross-sectional view (spine in circle, kidney in box).

**Fig. (2):**
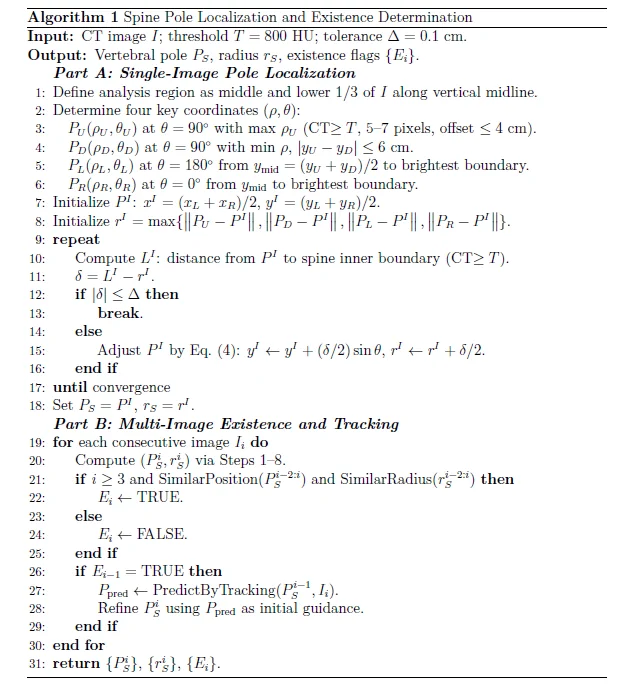
The complete procedure of the spine pole localization and existence determination.

**Fig. (3):**
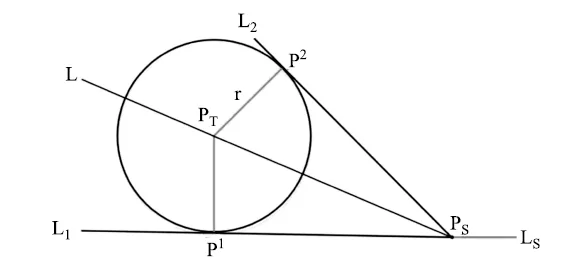
Schematic diagram of spinal pole *P_S_* and renal pole *P_T_*.

**Fig. (4):**
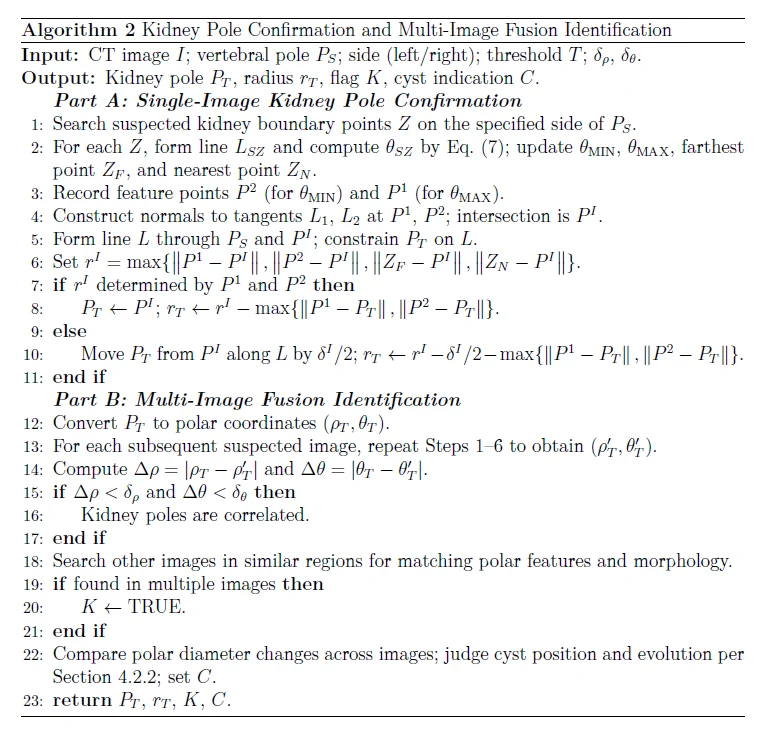
The complete procedure of the kidney pole confirmation and multi-image fusion identification.

**Fig. (5):**
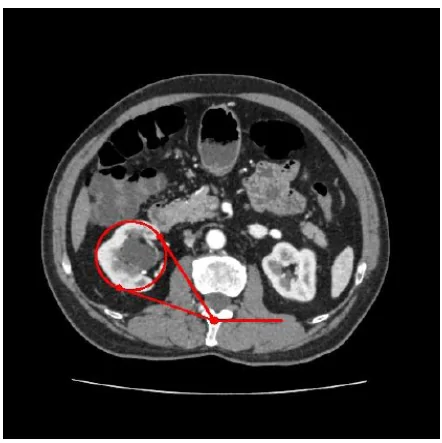
Schematic diagram of the dual-polar coordinate calibration of the shape of the spine and kidneys.

**Fig. (6):**
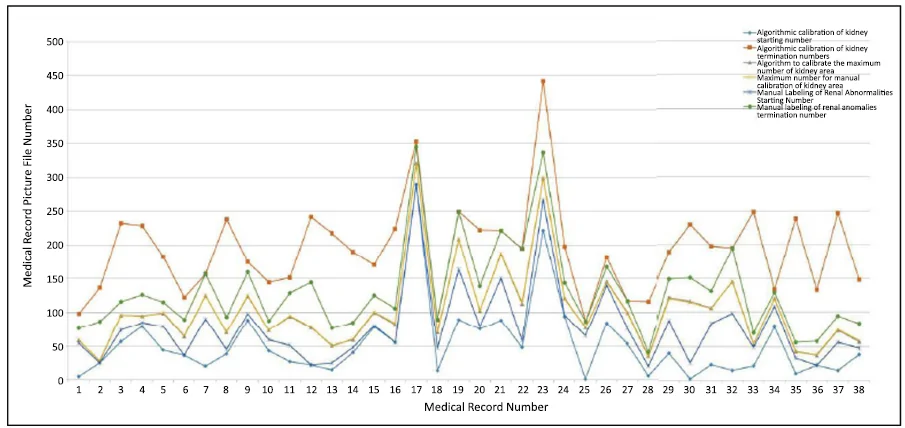
Schematic illustration of the locations of the starting points of the kidney and renal cysts.

## Data Availability

The datasets analyzed in the current work are not publicly available due to the need for follow-up research, but are available from the corresponding author [X.D] upon reasonable request.

## LIST OF ABBREVIATIONS

CT: Computed Tomography
OTSU: Otsu's threshold segmentation algorithm
MRI: Magnetic Resonance Imaging
AI: Artificial Intelligence
RCC: Renal Cell Carcinoma
3D: Three-dimensional
